# Lignin-Derived Hierarchical Porous Solid Base for Efficient Glucose Isomerization via In Situ Active Site Generation

**DOI:** 10.3390/ma19102112

**Published:** 2026-05-17

**Authors:** Mengqing Yang, Jun Xu, Peng Song, Ao Li, Maowang Zou, Shengtao Zhou

**Affiliations:** 1 State Key Laboratory of Advanced Papermaking and Paper-based Materials, Plant Fiber Materials Science Research Center, South China University of Technology, Guangzhou 510640, China; 202420128926@mail.scut.edu.cn (M.Y.); aoli.jul@gmail.com (A.L.); 202321029326@mail.scut.edu.cn (M.Z.); listzhou@mail.scut.edu.cn (S.Z.); 2 Nine Dragons Paper (Holdings) Limited, Dongguan 523000, China; spooknight@163.com

**Keywords:** lignin, biomass-derived carbon, solid alkali, glucose isomerization

## Abstract

Conventional biochar-based solid base catalysts often suffer from cumbersome preparation procedures and pore blockage during the loading of active components. To overcome these limitations, we developed an in situ construction strategy to fabricate hierarchically porous solid-base catalysts via cross-linking and carbonization of alkali lignin. Using alkali lignin as the carbon precursor, a soft-template-assisted cross-linking system enables the simultaneous formation of a hierarchical carbon framework and in situ generation of basic active sites through one-step pyrolysis under alkaline conditions. The physicochemical properties of the catalysts, including specific surface area, pore structure, and surface basicity, are effectively tuned by adjusting the carbonization temperature (600–800 °C). The optimized catalyst, KLPF-800, exhibits a high specific surface area of 309 m^2^·g^−1^ and a well-developed hierarchical pore architecture, delivering excellent catalytic performance in aqueous-phase glucose isomerization. A fructose yield of 33.21% is achieved at 120 °C within 20 min. This work provides a feasible strategy for valorizing lignin and designing efficient heterogeneous base catalysts.

## 1. Introduction

Faced with the increasingly severe energy crisis and environmental issues, the development of sustainable chemical production routes based on renewable biomass resources has become an important research direction [1,2,3]. As an abundant renewable carbon source, lignocellulose enables the high-value conversion of its derived platform molecule glucose, which is a crucial pathway for constructing a green chemical system. Among these transformations, the isomerization of glucose to fructose followed by further dehydration to produce 5-hydroxymethylfurfural (5-HMF) serves as a key route for obtaining furan-based polymers, fine chemicals, and biofuels [4,5,6]. However, the glucose isomerization process is limited by high reaction energy barriers and complex reaction pathways, leading to slow kinetics that typically act as the rate-determining step for subsequent conversions. Therefore, the development of efficient and cost-effective catalytic systems is of great significance [7].

Currently, glucose isomerization mainly relies on two pathways: enzymatic catalysis and chemical catalysis. Enzymatic catalysis has been industrialized, but it still suffers from high cost, harsh catalytic conditions, non-reusability, and short operational lifetime of enzymes [8]. In contrast, chemical catalytic systems, especially heterogeneous solid base catalysts, have attracted considerable attention due to their advantages of easy separation and environmental friendliness [9,10,11,12,13]. Relevant studies have shown that glucose isomerization generally follows the Lobry de Bruyn–van Ekenstein (LBvE) mechanism, whose core lies in the promotion of the formation and transformation of 1,2-enediol intermediates by basic sites [14,15]. In such catalytic systems, the performance strongly depends on the synergistic effect between the porous structure and basic active sites. A rational pore size distribution and large specific surface area facilitate the exposure of more active sites and promote mass transfer, while the spatial distribution and accessibility of active sites directly affect catalytic efficiency [16,17]. For example, hierarchically porous Sn-MFI zeolite exhibits a higher fructose yield than conventional Sn-BEA zeolite under the same glucose conversion [18]. Improving structural accessibility can significantly enhance catalytic efficiency, with a fructose yield of 25 mol% achieved under aqueous reaction at 120 °C for 5 min [19]. By constructing mesopores in single-crystal zeolites and confining In_2_O_3_ nanoparticles, not only spatial separation of basic and acidic sites was realized, but also a fructose yield of 54.9% was obtained under high glucose concentration [20]. Nevertheless, in practical catalyst design, the core challenge restricting further performance improvement remains: how to introduce basic active components while maintaining the stability of the porous structure, and achieve high dispersion and high accessibility of active sites simultaneously. For traditional supports such as zeolites and hydrotalcites, their ordered pore structures tend to collapse or block during impregnation or ion exchange, resulting in restricted mass transfer and reduced utilization efficiency of active sites [21]. Therefore, the development of novel support materials with both structural stability and tunable porous architecture is crucial to boosting catalytic performance.

Carbon-based materials, particularly biomass-derived porous carbon materials, exhibit great potential in catalysis owing to their excellent acid and alkali resistance, thermal stability, and tunable pore structure [22,23]. Among them, lignin, as the second most abundant natural polymer after cellulose, is rich in phenolic hydroxyl and aliphatic hydroxyl groups. It not only provides abundant reactive sites for the structural regulation of carbon materials, but also serves as an ideal precursor for the construction of functionalized porous carbon materials [24,25,26,27]. Previous studies have demonstrated that biomass-based carbon materials can act as efficient catalytic supports for glucose isomerization. Kang et al. [28] sequentially impregnated MgCl_2_, AlCl_3_ and KHCO_3_ onto cellulose-derived carbon supports, followed by secondary pyrolysis, to prepare a biochar-based catalyst with multiple catalytically active sites. A fructose yield of 38.7% was achieved in aqueous phase at 100 °C for 30 min. Similarly, Zhang et al. [29] employed cellulose as the carbon precursor to fabricate an Al-doped hydrochar catalyst with hierarchical pore structure via activation with KHCO_3_ and K_2_CO_3_, affording a fructose yield of 32.6% under aqueous reaction at 160 °C for 20 min. Nevertheless, the preparation of most existing biochar-based catalysts still follows a multi-step strategy of “carbonization first, followed by active site introduction”. Specifically, biomass is first converted into carbon materials via pyrolysis or hydrothermal carbonization, and then catalytically active components are introduced through secondary carbonization, impregnation, or activation. Although such approaches enable the construction of active sites, they are often accompanied by pore blockage, reduced specific surface area, and restricted mass transfer efficiency, while increasing the energy consumption and complexity of the preparation process. Therefore, it remains challenging to achieve synergistic regulation of pore structure construction and basic active site introduction without causing structural damage.

Here, we develop an in situ integrated cross-linking–carbonization strategy to fabricate lignin-based solid-base catalysts with a hierarchical porous structure. Specifically, alkali lignin was employed as the carbon precursor, while polyethylene glycol diglycidyl ether was used to construct a stable three-dimensional cross-linked network, and Pluronic F127 was introduced as a soft template to regulate the formation of porous structure. Notably, a small amount of sodium hydroxide added during preparation not only acts as a reaction medium to promote the cross-linking reaction, but also exerts dual functions synchronously during the subsequent carbonization: on one hand, it creates micropore and mesoporous structures through alkali etching; on the other hand, it realizes the in situ doping of alkali metal species within the carbon matrix, thereby forming stable basic active sites. Compared with the traditional multi-step “support preparation–active component loading” method, this strategy enables the simultaneous regulation of porous structure construction and active site introduction, which prevents structural collapse and simultaneously improves the dispersion and accessibility of active sites. By systematically tuning the carbonization temperature, combined with scanning electron microscopy, nitrogen adsorption–desorption analysis, Raman spectroscopy, and high-performance liquid chromatography, the structural characteristics of the catalysts and their catalytic performance for glucose isomerization were systematically investigated, and the structure-activity relationship among pore structure, surface chemical properties, and catalytic performance was further revealed. This study provides a simple, effective, and novel strategy for the high-value utilization of lignin and the rational design of high-performance solid base catalysts.

## 2. Materials and Methods

### 2.1. Experimental Reagents and Instruments

Experimental reagents: Alkali lignin was supplied by Shandong Huatai Paper Co., Ltd. (Dongying, China) Pluronic F127 (Mₙ ≈ 13 000), polyethylene glycol diglycidyl ether (PEGDGE), sodium hydroxide (NaOH), glucose (purity ≥ 99.5%), and fructose (purity ≥ 99.5%) were all purchased from Shanghai Macklin Biochemical Technology Co., Ltd. (Shanghai, China). All reagents were of analytical grade and used as received without further purification.

Experimental instruments: The main instruments used in this study included a three-zone tube furnace (OTF-1200X-III, Hefei Kejing Materials Technology Co., Ltd., Hefei, China), an electric thermostatic drying oven (DHG-9423A, Shanghai Jinghong Experimental Equipment Co., Ltd., Shanghai, China), a hot plate (HP-30D, Daihan Scientific, South Korea), and an ultrasonic cleaner (SUC-6H, Shanghai Titan Technology Co., Ltd., Shanghai, China).

### 2.2. Preparation of Lignin-Derived Hierarchically Porous Solid Base

**Figure 1:**
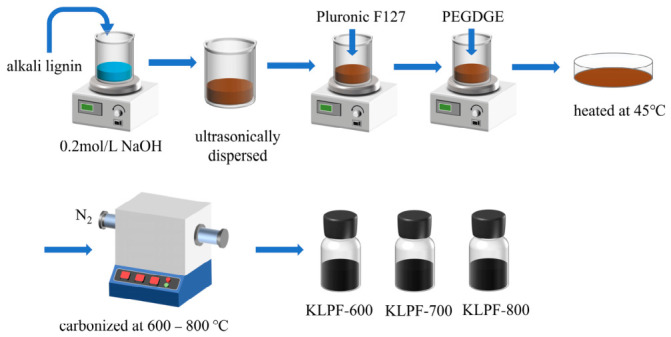
Schematic procedure for the preparation of solid-base catalysts.

10 g of oven-dried alkali lignin was dissolved in 0.2 mol·L^−1^ NaOH solution, and a uniform dispersion was obtained after 30 min of ultrasonic treatment. Under continuous stirring, Pluronic F127 with an equal mass to alkali lignin was added into the above solution, and the stirring was continued for 1 h. Subsequently, 4 mL of PEGDGE was added dropwise, followed by continuous stirring for 1 h to complete the cross-linking reaction. The final homogeneous mixture was transferred into a Petri dish and dried at 45 °C on a hot plate for 24 h to obtain a black viscous alkali precursor film. The precursor was placed in a tube furnace and heated at a rate of 5 °C·min^−1^ under a nitrogen atmosphere, followed by isothermal carbonization at 600 °C, 700 °C, and 800 °C for 2 h, respectively. After naturally cooling to room temperature, a series of samples were obtained and labeled as KLPF-600, KLPF-700, and KLPF-800. In addition, the sample prepared by direct carbonization of pure alkali lignin at 600 °C for 2 h was denoted as KL. The experimental procedure is shown in Figure 1.

### 2.3. Characterization of Solid Base Catalysts

The surface morphology and microstructure of the as-prepared samples KLPF-600, KLPF-700, and KLPF-800 were observed using a Meriel field-emission scanning electron microscope (SEM, Zeiss, Oberkochen, Germany) with an electron acceleration voltage set to 5 kV. Nitrogen adsorption–desorption measurements were performed on an automated surface area and pore size analyzer (Micromeritics ASAP 2460, Norcross, GA, USA) at 77 K under liquid nitrogen temperature. The specific surface area of the samples was calculated using the Brunauer–Emmett–Teller (BET) model, while the pore size distribution and pore diameter were analyzed by the density functional theory (DFT) method. All samples were degassed in vacuum at 200 °C for more than 8 h prior to measurement to remove surface-adsorbed species. A confocal laser micropore Raman spectrometer (Horiba Jobin Yvon LabRAM Aramis, Palaiseau, France) was employed to characterize the graphitization degree and defect structure of the carbon materials over a wavenumber range of 500–4000 cm^−1^. The crystalline structure of the samples was analyzed by X-ray diffraction (XRD, PANalytical X’Pert3 Powder, Almelo, Netherlands) with Cu Kα radiation, operating at 40 kV and 40 mA over a scanning range of 2θ = 5–80° with a step size of 0.02°. Fourier transform infrared (FT-IR) spectra were recorded on a Bruker VERTEX 70 spectrometer (Bruker, Berlin, Germany) in the range of 500–4000 cm^−1^ using the KBr pellet method. Typically, 1 mg of sample was thoroughly mixed and ground with 100 mg of spectroscopic-grade potassium bromide (KBr) in an agate mortar and then pressed into a transparent pellet. Finally, X-ray photoelectron spectroscopy (XPS, Kratos Axis Supra+, Southampton, UK) with Al Kα radiation was used to qualitatively and quantitatively analyze the surface elemental composition and chemical valence states of the samples.

### 2.4. Catalytic Performance Evaluation

The catalytic performance of the as-prepared solid base catalysts for the isomerization of glucose to fructose was investigated in an aqueous system. 0–70 mg of catalyst was added into the reactor, followed by the injection of 5 mL of 50 mg·mL^−1^ aqueous glucose solution as the reactant. The reactions were carried out at temperatures of 80–140 °C for 20–70 min. The effects of reaction parameters including reaction temperature, reaction time, and catalyst dosage on glucose isomerization were systematically studied. After the reaction, the reactor was cooled to room temperature, and the supernatant was withdrawn with a syringe and filtered through a 0.22 μm syringe filter for subsequent liquid chromatography analysis.

Fructose content was qualitatively and quantitatively analyzed using high-performance liquid chromatography (HPLC, Agilent 1260, USA). A 5 mM H_2_SO_4_ solution was used as the mobile phase with a flow rate of 0.6 mL·min^−1^. The injection volume was 20 μL, the column oven temperature was set at 55 °C, and the detector temperature was 35 °C. The retention times of glucose and fructose were around 15 min. The formulas for calculating glucose conversion, fructose selectivity, and fructose yield are as follows:

$$Glucose\;Conversion(\%)=\frac{nGlucose,initial-nGlucose,remaining}{nGlucose,initial}\times 100$$

$$Fructose\;Selectivity(\%)=\frac{nFructose,formed}{nGlucose,initial-nGlucose,remaining}\times 100$$

$$Fructose\;Yield(\%)=\frac{nFructose,formed}{nGlucose,initial}\times 100$$

## 3. Results and Discussion

### 3.1. Structural Characterization of Lignin-Based Solid Base Catalysts

#### 3.1.1. Morphological Analysis of the Catalyst

**Figure 2:**
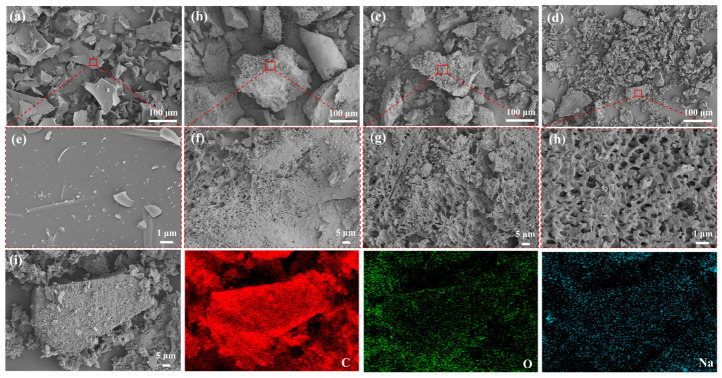
SEM images of (**a**) KL, (**b**) KLPF-600, (**c**) KLPF-700, and (**d**) KLPF-800; high-resolution SEM images of (**e**) KL, (**f**) KLPF-600, (**g**) KLPF-700, and (**h**) KLPF-800; (**i**) EDS spectrum of KLPF-800.

SEM was employed to directly observe the apparent morphology, particle size and dispersion of the catalyst, as well as to analyze its pore structure and surface defects, thereby providing direct evidence for revealing the structure-activity relationship between the material microstructure and catalytic performance, the results are shown in Figure 2. As can be seen from the SEM images in Figure 2a–c, the pristine alkali lignin without cross-linking exhibits sharp-edged, dense blocky agglomerates with a smooth surface and negligible porosity after carbonization at 600 °C. After introducing PEGDGE to construct a three-dimensional cross-linked network combined with F127 soft template regulation, the morphology of the KLPF series samples evolves continuously with increasing carbonization temperature: at 600 °C (KLPF-600), the blocky particles fragment into rough-surfaced irregular aggregates with sparse and unevenly sized pores; after carbonization at 700 °C (KLPF-700), the proportion of porous regions expands significantly and the number of channels increases; when raised to 800 °C (KLPF-800), the material presents a high-density, interconnected hierarchical pore structure with a more uniform pore size distribution, which is mainly attributed to the contraction of the carbon network and complete removal of the template at high temperature, greatly increasing the specific surface area. Additionally, the apparent pore size of KLPF-800 was further analyzed quantitatively, and the corresponding pore size distribution curve is shown in Figure S1a. Although the pore structures formed under different carbonization temperatures vary, a continuous and unobstructed pore system can be observed in all samples, which effectively avoids pore blockage caused by the secondary loading of active sites in conventional methods. This facilitates sufficient contact between reactant molecules and active sites, as well as the rapid desorption and diffusion of products.

In addition, Figure S1b,c, shows that the sample without PEGDGE has fewer surface pores after carbonization, and its pore structure differs from that of the KLPF series materials, where pore formation is primarily related to the escape of the template during high-temperature carbonization. This indicates that the porous structure of the carbonized material mainly stems from the continuous, interconnected porous cross-linked network formed among alkali lignin, PEGDGE, and Pluronic F127. Without this cross-linked network, the F127 soft template alone cannot effectively exert its pore-forming effect, further confirming that the materials prepared by the cross-linking method possess abundant pore channels and good pore connectivity, which are beneficial for catalytic activity. Furthermore, the EDS spectrum in Figure 2i, demonstrates that the surface of the as-prepared KLPF-800 sample contains C, O, and Na elements, with Na and C elements highly co-localized without obvious agglomeration and uniformly distributed on the material surface. This achieves the synergistic regulation of hierarchical porous structure construction and active site introduction, verifying the successful preparation of lignin-derived hierarchically porous solid base catalysts.

#### 3.1.2. Specific Surface Area and Porosity Analysis

**Figure 3:**
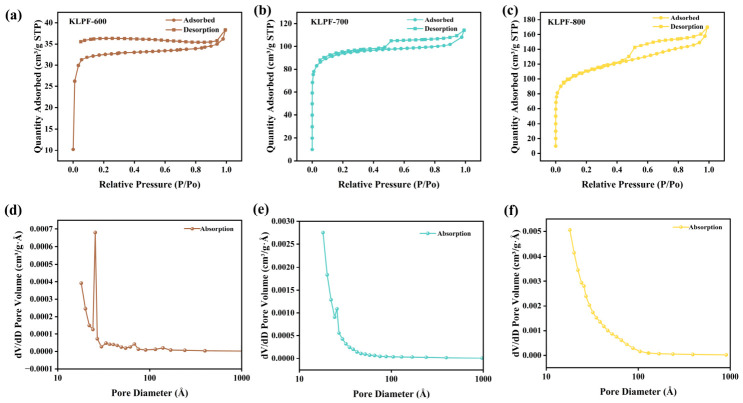
(**a**–**c**) N_2_ adsorption–desorption isotherms of the samples; (**d**–**f**) corresponding DFT pore size distributions of the samples.

The pore structure and specific surface area of biomass-derived carbon materials are crucial to their catalytic performance [16]. The pore structures of the samples were analyzed by N_2_ adsorption–desorption measurements. Figure 3a–c, display the adsorption–desorption isotherms of KLPF materials at different carbonization temperatures. All three samples exhibit typical type IV isotherms. The rapid N_2_ adsorption at low relative pressure (close to 0) indicates the presence of abundant micropores, while distinct hysteresis loops appear at high relative pressure (P/P_0_ > 0.8), confirming the coexistence of micropore and mesoporous hierarchical structures and good pore connectivity within the materials [30,31]. In addition, the corresponding DFT pore size distribution curves in Figure 3d–f further reveal that the pore sizes of the KLPF series materials are mainly concentrated in the mesoporous range (<10 nm), with an obvious distribution peak at approximately 5 nm, verifying the successful construction of hierarchical porous structures. With increasing temperature, the pore size distribution peak gradually broadens and shifts slightly toward larger pores, accompanied by a continuous increase in the pore volume peak. KLPF-800 shows the most abundant pore volume distribution, which is highly consistent with the adsorption isotherm results. This result fully demonstrates that the network structure formed by cross-linking provides rigid support for the pore framework. After high-temperature carbonization, KLPF materials achieve a hierarchical porous structure dominated by mesopores with supplementary micropores. Such a hierarchical pore structure can effectively improve catalytic performance: specifically, micropores can provide abundant active sites, while mesopores promote mass transfer and liquid diffusion, thereby accelerating the reaction rate.

The specific surface area (S_BET_), total pore volume (V_tot_), and average pore width (PW) of the materials at different carbonization temperatures were calculated using the BET model, and these values show a clear temperature-dependent trend. As shown in Table 1, increasing the carbonization temperature from 600 °C to 800 °C causes S_BET_ to rise sharply from 128 m^2^·g^−1^ to 309 m^2^·g^−1^, which clearly indicates that high-temperature carbonization effectively induces reconstruction of the carbon framework and full development of the pore structure. Meanwhile, the pore volume of KLPF-800 is slightly lower than that of KLPF-700, suggesting that partial collapse of fragile pore walls at high temperature leads to a decrease in total pore volume. At the same time, PW gradually increases from 18 Å to 34 Å, reflecting that high temperature not only increases the number of pores but also promotes coarsening and interconnection of the pore structure. This may be attributed to the merging of adjacent small pores into larger ones, which increases the average pore size. Although this process reduces part of the pore volume, it improves pore connectivity and size. The high specific surface area and hierarchical porous structure obtained at 800 °C facilitate molecular mass transfer and uniform distribution of active sites, thus enhancing catalytic efficiency.

#### 3.1.3. Chemical Structure Analysis of the Catalyst

**Figure 4:**
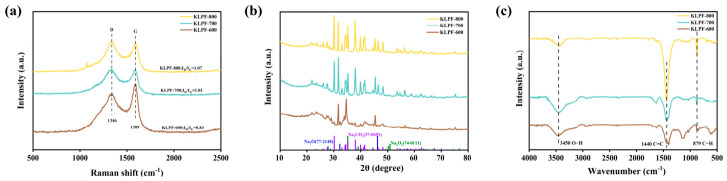
(**a**) Raman spectra; (**b**) XRD pattern; (**c**) FT-IR spectra of the KLPF materials.

Raman spectroscopy is employed to analyze the graphitization degree, defect structure and surface functional group characteristics of carbon materials. The intensity ratio of characteristic peaks (I_D_/I_G_) can be used to effectively evaluate the disorder degree and defect density of the carbon framework, providing structural evidence for clarifying the structure-activity relationship among pore structure, defect sites and catalytic performance. The Raman spectra of KLPF samples at different carbonization temperatures are displayed in Figure 4a. All samples exhibit two broad bands: the D band at approximately 1346 cm^−1^ and the G band at around 1589 cm^−1^ [32,33]. These two bands correspond to the in-plane stretching vibration of disorder/defect structures at the edges of carbon crystallites and the ordered sp^2^-hybridized graphitic structure, respectively. The intensity ratio of the D peak to the G peak (I_D_/I_G_) is generally used to evaluate the disorder degree of carbon materials [34]. As the carbonization temperature increases from 600 °C to 800 °C, the I_D_/I_G_ ratio of KLPF materials rises from 0.83 to 1.07, indicating that the elevated carbonization temperature significantly increases the defect density and the number of edge carbon active sites on the material surface. This can be attributed to the accelerated gas release rate and enhanced etching reaction rate and extent of sodium hydroxide at higher carbonization temperatures, which lead to a more developed pore structure and more newly exposed carbon edges, thus resulting in an increased I_D_/I_G_ ratio.

XRD is used to analyze the phase composition, crystal structure and crystallinity of the catalyst, determine the existing form and dispersion of active components, identify characteristic diffraction peaks, and verify the successful preparation of the catalyst. As shown in Figure 4b, it can be observed that all three curves exhibit a broad diffuse peak near 2θ ≈ 26°, corresponding to the (002) plane of amorphous carbon [35,36]. With increasing carbonization temperature, the peak intensity gradually decreases, indicating an increase in the disorder degree of porous carbon at high temperatures, which is consistent with the Raman results.

In addition, several sharp diffraction peaks can be clearly observed in the range of 2θ = 20–40°, and the peak positions match well with the crystalline phases of sodium-containing species such as Na_2_O (PDF#77-2148), Na_2_CO_3_ (PDF#37-0451), and Na_2_O_2_ (PDF#74-0111) marked below. Moreover, the intensity of these diffraction peaks is significantly enhanced as the carbonization temperature rises from 600 °C to 800 °C. These sodium species are the key components constituting the surface basic active sites, derived from the small amount of reaction medium (sodium hydroxide) introduced to promote the formation of the cross-linking network, which is transformed during the subsequent high-temperature carbonization process. The high-temperature environment not only strengthens the crystalline structure of sodium species but also facilitates their uniform dispersion and stable existence on the carbon framework. Such structural characteristics, in which highly crystalline sodium species and abundant basic sites coexist formed during high-temperature carbonization, provide sufficient active centers for the solid base catalyst.

FT-IR is employed to identify the types of functional groups, chemical bonding states, analyze the interaction between the carbon support and basic sites, and provide chemical evidence for the structural composition of the catalyst. As shown in Figure 4c, the absorption peaks of the materials at 3450 cm^−1^, 1440 cm^−1^, and 879 cm^−1^ correspond to -OH groups, C=C stretching vibrations arising from aromatization of the carbon framework, and C-H out-of-plane bending vibrations of aromatic rings, respectively [37,38]. As the carbonization temperature increases from 600 °C to 800 °C, the C=C absorption peak at 1440 cm^−1^ is significantly enhanced, indicating an improved degree of aromatization in the carbon skeleton. This peak is also related to the asymmetric stretching vibration of CO_3_^2−^, and its increased intensity suggests that the content of sodium-containing active phases rises with higher carbonization temperature, providing more abundant basic catalytic sites for the material [39]. This phenomenon indicates that high-temperature carbonization not only promotes the deep reconstruction of the carbon framework but also facilitates the formation and enrichment of surface functional groups, which is consistent with the XRD results.

#### 3.1.4. XPS Analysis

**Figure 5:**
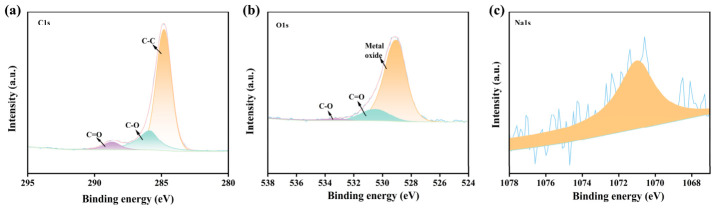
High-resolution (**a**) C 1s, (**b**) O 1s, and (**c**) Na 1s spectra of KLPF-800.

XPS clearly reveals the surface chemical composition and elemental valence state characteristics of the KLPF series solid base catalysts. As shown in Figure S2, the full XPS spectrum of KLPF-800 exhibits three characteristic peaks at 284 eV, 529 eV, and 1071 eV, corresponding to C, O, and Na elements, respectively. Furthermore, peak fitting was performed for C 1s, O 1s, and Na 1s spectra. As seen in the C 1s spectrum (Figure 5a), the peaks at 284.79 eV, 285.86 eV, and 288.64 eV are assigned to C-C, C-O, and C=O bonds, respectively. Among them, C=C constitutes the main structure of the carbon framework, while the presence of oxygen-containing functional groups (C-O and C=O) confirms that the material surface is rich in polar active sites. The O 1s spectrum (Figure 5b) can be further deconvoluted into metal oxide (~529.0 eV), C=O (~530.47 eV), and C-O (~533.23 eV). The metal oxide peak corresponds to sodium-based basic species, which are the key components of the catalytic active centers in the solid base catalyst. The Na 1s spectrum (Figure 5c) shows a typical sodium compound characteristic peak at 1071 eV, indicating that sodium species are uniformly dispersed on the carbon support in the form of basic oxides and carbonates, verifying the successful introduction of basic sites. These results demonstrate that the as-prepared KLPF-800 catalyst not only possesses abundant oxygen-containing functional groups to enhance surface polarity and substrate adsorption capacity, but also successfully introduces highly dispersed sodium-based basic active sites. The synergistic effect of these two features constructs a highly efficient solid base catalytic interface, providing sufficient active sites and a favorable chemical environment for substrate activation and conversion during the catalytic reaction

### 3.2. Performance Evaluation of Lignin-Based Solid Base Catalysts

#### 3.2.1. Effects of Isomerization Reaction Temperature and Time

**Figure 6:**
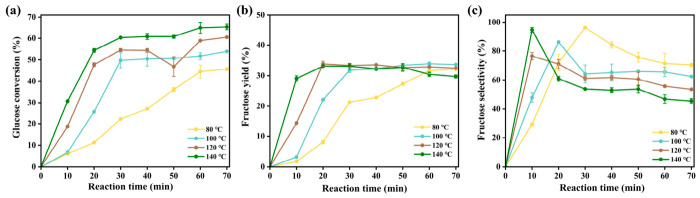
(**a**) Glucose conversion, (**b**) fructose yield, and (**c**) fructose selectivity of KLPF-800 under varied reaction temperature and time.

On the basis of the above characterization results, KLPF-800 was selected as the candidate material for further investigation of the isomerization process conditions, mainly owing to its large specific surface area and abundant basic sites. The effects of reaction temperature and time on its catalytic activity are presented in Figure 6. As shown in Figure 6b, fructose yield increased markedly from 7.69% to 33.21% when the isomerization temperature was raised from 80 °C to 120 °C. However, further increasing the temperature to 140 °C caused the yield to decrease to 29.41% after 70 min. Figure 6a shows that glucose conversion is strongly temperature-dependent, gradually increasing over the 80–140 °C range. Under mild conditions (80 °C, 30 min), fructose selectivity reached 96.71%, likely due to the suppression of side reactions. At higher temperatures, selectivity declined, probably as a result of by-product formation, including humins and small-molecule organic acids such as formic and lactic acids [40,41]. The effect of reaction time was also examined at different temperatures. Glucose conversion increased with reaction time from 0 to 70 min at all temperatures, though the rate of increase slowed and eventually plateaued (Figure 6a). Under high-temperature conditions, fructose yield tended to decrease with prolonged reaction (Figure 6b). Specifically, the maximum yield of 33.6% was observed at 120 °C for 30 min, while extending the reaction time from 20 min to 30 min resulted in only a slight increase. Considering energy efficiency, 120 °C and 20 min were selected as the optimal conditions, balancing high fructose yield, sufficient glucose conversion, and minimized side reactions.

#### 3.2.2. Effect of Catalyst Dosage

**Figure 7:**
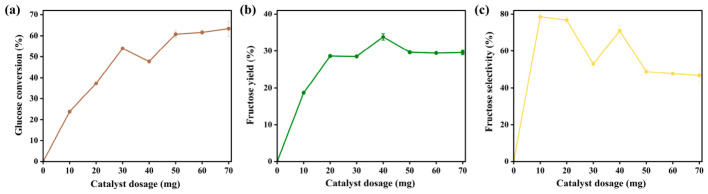
(**a**) Glucose conversion, (**b**) fructose yield, and (**c**) fructose selectivity of KLPF-800 under different catalyst loadings at 120 °C and 20 min.

The effect of catalyst dosage on the reaction was investigated at 120 °C for 20 min, with dosages ranging from 0 to 70 mg. As shown in Figure 7a, glucose conversion increased steadily and significantly with increasing catalyst dosage, indicating that higher dosages provide more active sites and thus enhance glucose conversion. However, at low catalyst loadings (0–30 mg), fructose yield remained low due to insufficient active sites, resulting in incomplete isomerization under the fixed reaction conditions. Notably, the fructose yield reached a maximum of 33.21% at a catalyst dosage of 40 mg. Beyond this dosage, the yield gradually decreased, dropping to 29.13% at 70 mg, which is consistent with the fructose selectivity trends shown in Figure 7c. At 10 mg, fructose selectivity was 78.4%, but it declined continuously as the catalyst dosage increased. The reduction in both yield and selectivity at high catalyst loadings can be attributed to excessive active sites, which accelerate side reactions such as condensation and rehydration under low activation energy conditions [15]. Considering glucose conversion, fructose yield, and selectivity together, 40 mg was determined to be the optimal catalyst dosage, providing the highest fructose yield while maintaining relatively high selectivity and sufficient glucose conversion.

#### 3.2.3. Catalyst Stability Analysis

**Figure 8:**
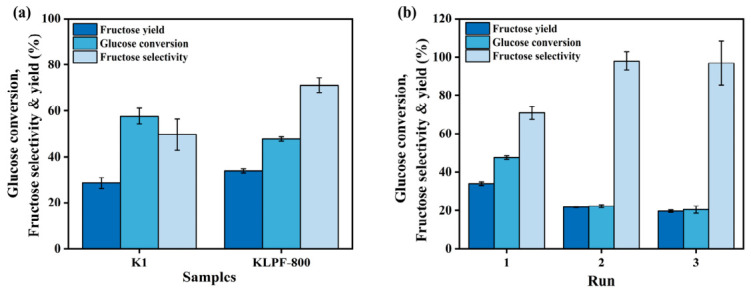
(**a**) Performance of KLPF-800 vs. water-pretreated K1 in glucose isomerization. (**b**) Reusability test of 40 mg KLPF-800 at 120 °C for 20 min.

To evaluate the stability of the basic sites under high-temperature aqueous conditions, the KLPF-800 sample was subjected to a hydrothermal treatment. Specifically, 40 mg of KLPF-800 was added to 5 mL of pure water in a PTFE-lined autoclave and heated at 120 °C for 20 min. After cooling, the catalyst was recovered by centrifugation and denoted as K1. For the catalytic test, 5 mL of a 50 mg·mL^−1^ glucose solution and the pre-treated catalyst K1 were added to a PTFE-lined autoclave, and the reaction was carried out under the same conditions. The catalytic performance of K1 was then compared with that of the fresh KLPF-800 catalyst (40 mg) used directly under identical conditions. The results are shown in Figure 8a. As illustrated in Figure 8a, after high-temperature water immersion, the fructose yield decreased slightly, while glucose conversion showed a minor increase. The fructose selectivity decreased from approximately 70% to 50%. These results indicate that, although minor leaching of basic sites occurs, the majority of the sodium-based active sites remain intact, demonstrating that the catalyst maintains a certain degree of stability under high-temperature aqueous conditions.

Additionally, the reusability of the KLPF-800 catalyst was evaluated under optimal reaction conditions over three consecutive runs, as shown in Figure 8b. After three cycles, the fructose yield decreased from approximately 33% to 19%, with the most significant decline occurring after the first cycle. This behavior can be primarily attributed to the fact that the active sites of solid-base catalysts are mainly derived from metal oxides, a challenge also observed in other studies on carbon-supported metal oxide catalysts for glucose isomerization [42,43]. According to previous reports, the loss of active metal oxide sites is mainly caused by the formation of acidic byproducts during glucose isomerization and the deposition of insoluble humic-like substances that block the pores of the catalyst, thereby hindering substrate access [44,45]. Notably, the catalytic performance stabilized after the second and third cycles, which may be ascribed to the combined effects of interactions between sodium species and the mesoporous carbon support, as well as the confinement effect of the hierarchical carbon structure [10,41]. These results suggest that, despite initial leaching or surface blockage, the majority of the solid-base active sites remain preserved, and the catalyst exhibits a certain degree of stability during repeated use.

#### 3.2.4. Catalyst Performance Evaluation

To evaluate the catalytic performance of KLPF-800 in the isomerization of glucose to fructose, a comparative analysis was conducted with solid-base catalysts reported in the previous literature, and the results are summarized in Table 2. As shown, KLPF-800 exhibits a superior fructose yield of 33.21% under mild and time-efficient conditions (120 °C, 20 min), outperforming most of the reported heterogeneous catalysts. KLPF-800 achieves a significantly higher fructose yield with a much shorter reaction time, demonstrating excellent catalytic activity and reaction kinetics. Even when compared to the high-yielding Mg-C_3_N_4_ catalyst (32.50% yield), KLPF-800 delivers a comparable or slightly higher yield while requiring only 1/12 of the reaction time (20 min vs. 240 min), which greatly enhances the energy efficiency. The outstanding performance of KLPF-800 can be attributed to its unique structural and compositional advantages derived from its preparation method. Unlike conventional solid-base catalysts that rely on expensive metal precursors, or complex synthesis procedures, KLPF-800 is fabricated from renewable lignin-based raw materials via a simple, green crosslinking and carbonization strategy. This synthetic route not only reduces production costs and environmental impact but also endows the catalyst with a hierarchical porous structure, abundant surface basic sites, and excellent mass transfer efficiency. In summary, KLPF-800 stands out as a promising heterogeneous catalyst for glucose isomerization, which provides a green and efficient alternative for the industrial production of high-fructose syrup.

## 4. Conclusions

In this work, aiming at the efficient and economical catalysis for glucose isomerization, a series of hierarchical porous solid-base catalysts (KLPF series) derived from industrial alkali lignin were fabricated, and the effects of carbonization temperature on the structure and catalytic performance were systematically investigated. The results demonstrate that carbonization temperature significantly regulates the porous structure and distribution of basic active sites. Among them, KLPF-800 prepared at 800 °C exhibits a high specific surface area of 309 m^2^g^−1^, a well-developed hierarchical pore structure, and abundant sodium-based basic sites, thus delivering the optimal catalytic performance. Under the conditions of 120 °C, 20 min, and a catalyst dosage of 40 mg, a fructose yield of 33.21% is achieved over KLPF-800, which is distinctly superior to most reported solid-base catalysts. The key to the enhanced performance lies in the synergistic effect between the hierarchical porous structure and basic active sites: the micropore and mesoporous structure not only facilitates the diffusion of reactant molecules and improves mass transfer efficiency, but the abundant and uniformly distributed basic sites also accelerate the isomerization rate from glucose to fructose. Such a synergistic regulation mechanism of “pore structure–active sites” enables the catalyst to maintain high efficiency even in high-concentration glucose systems and overcomes the pore collapse of conventional supports during the loading of active components. In summary, this study realizes high-value lignin valorization and provides a green, scalable solid-base catalyst strategy. Future efforts will optimize pore architecture and active phase loading to boost fructose yield and recyclability, facilitating industrial application in biomass platform chemical production.

## Figures and Tables

**Table 1:** Specific surface area and pore parameters of the KLPF series porous carbon materials.

Sample	S_BET_ (m^2^·g^−1^)	V_tot_ (m^3^·g^−1^)	PW (Å)
KLPF-600	128	0.05	18
KLPF-700	224	0.11	29
KLPF-800	309	0.09	34

**Table 2:** Catalytic performance of various heterogeneous catalysts for glucose isomerization in water.

Catalyst	Temperature (°C)	Time (min)	Fructose Yield (%)	References
BC-Al200	140	40	29.40	[46]
Mg-C_3_N_4_	90	240	32.50	[11]
PS-TBD	140	30	28	[47]
40%MgO/NbP-500	120	30	24.60	[48]
6Mg/NaX from cogon grass	100	20	25.8	[49]
MgO-Biochar	80	120	26.0	[50]
PEGDA-DMAPMA	110	120	27.0	[51]
KLPF-800	120	20	33.21%	This work

## Data Availability

The original contributions presented in this study are included in the article. Further inquiries can be directed to the corresponding author.

## Supplementary Materials

The following supporting information can be downloaded at: https://www.mdpi.com/article/10.3390/ma19102112/s1, Figure S1: (a) Pore size distribution of KLPF-800. (b) SEM and (c) high-resolution SEM images of the PEGDGE-free sample; Figure S2: XPS spectrum of KLPF-800
