# Supplementary material for: Lignin-Derived Hierarchical Porous Solid Base for Efficient Glucose Isomerization via In Situ Active Site Generation
Source: Materials (Basel). 2026 May 17;19(10):2112. doi: 10.3390/ma19102112 (PMC13208190; doi:10.3390/ma19102112)
Supplement: Supplementary file 1 [file materials-19-02112-s001.zip › materials-4295926-supplementary.pdf]

Supplementary Information

Lignin-Derived Hierarchical Porous Solid Base for Efficient Glucose Isomerization via In Situ Active Site Generation

Mengqing Yang[a], Jun Xu[a*], Peng Song[b], Ao Li[a], Maowang Zou[a], Shengtao Zhou[a]

[a] State Key Laboratory of Advanced Papermaking and Paper-based Materials, Plant Fiber Materials Science Research Center, South China University of Technology, Guangzhou 510640, China

[b] Nine Dragons Paper (Holdings) Limited, Dongguan, 523000, China

Corresponding author: Jun Xu

E-mail address: xujun@scut.edu.cn

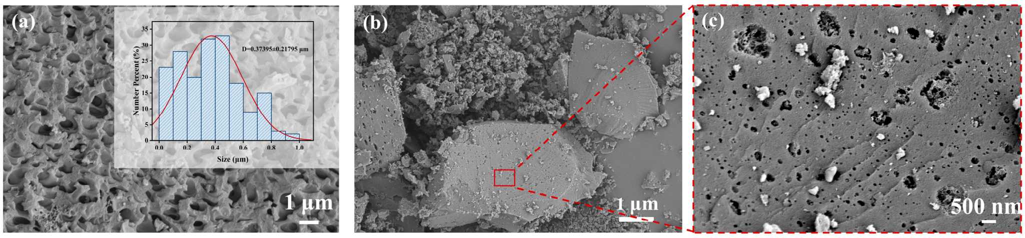

Figure S1.(a) Pore size distribution of KLPF-800. (b) SEM and (c) high-resolution SEM images of the PEGDGE-free sample

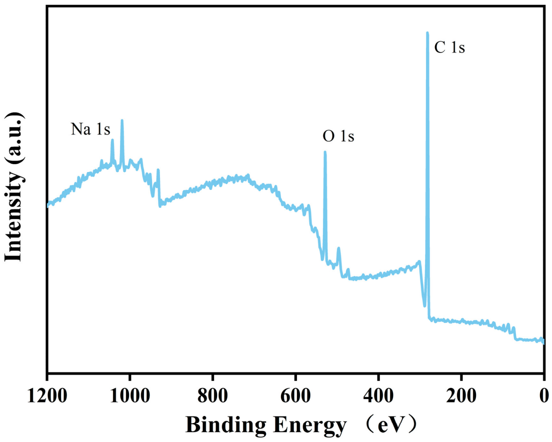

Figure S2. XPS spectrum of KLPF-800
